# Molecular Mechanisms of Cell-to-Cell Transmission in Human Herpesviruses

**DOI:** 10.3390/v17060742

**Published:** 2025-05-22

**Authors:** Liyuan Yan, Jing Guo, Yinan Zhong, Jiangbo Wei, Zejun Wang

**Affiliations:** 1 Wuhan Institute of Biological Products Co., Ltd., No. 1 Huangjin Industrial Park Road, Wuhan 430207, China; yanliyuan2000@163.com (L.Y.); guojingwh2004@hotmail.com (J.G.); 2 National Engineering Technology Research Center for Combined Vaccines, Wuhan 430207, China; 3 National Key Laboratory for Novel Vaccines Research and Development of Emerging Infectious Diseases, Wuhan 430207, China; 4 Hubei Provincial Vaccine Technology Innovation Center, Wuhan 430207, China; 5 Department of Pharmaceutical Engineering, School of Engineering, China Pharmaceutical University, Nanjing 210009, China; zhongyinan@126.com; 6 Weijiangbo Laboratory, National Vaccine and Serum Institute, Beijing 101111, China; weijiangbo@sinopharm.com

**Keywords:** human herpesvirus, cell-to-cell transmission, syncytium, tight junction, exosomes, tunneling nanotubes, envelope glycoproteins

## Abstract

Members of the family *Orthoherpesviridae* employs two distinct transmission modes: free virion release and cell-to-cell transmission. The latter enables immune evasion through multiple mechanisms, facilitating infections in skin, mucosa, and neural tissues. This review synthesizes current knowledge on human herpesvirus cell-to-cell transmission mechanisms, including syncytium formation, tight junction exploitation, exosomal transfer, and tunneling nanotube utilization. We analyze how these strategies enhance infection efficiency, evade immune surveillance, and augment pathogenicity. Furthermore, we discuss recent intervention strategies targeting cell-to-cell transmission, including the development of monoclonal antibodies, antiviral drugs, and vaccines. These inights provide a theoretical foundation for developing novel approaches against human herpesvirus infections.

## 1. Introduction

Human herpesviruses, belonging to the family *Orthoherpesviridae,* comprises large, enveloped double-stranded DNA viruses and is classified into three subfamilies based on biological and genetic characteristics (Table 1) [1]. With global seroprevalence rates exceeding 60–90%, these pathogens cause significant morbidity through the infections of cutaneous, mucosal, and neurological tissues, posing a serious threat to human health [2]. Human herpesviruses employ dual dissemination strategies: classical cell-free virion release and direct cell-to-cell transmission. The latter mechanism facilitates immune evasion by minimizing viral exposure to extracellular immune surveillance. Understanding these transmission mechanisms is crucial for developing targeted therapeutic strategies. This review examines recent advances in human herpesvirus cell-to-cell transmission mechanisms and explores their implications for antiviral development (Table 2).

## 2. Modes of Human Herpesvirus Cell-to-Cell Transmission

**Figure 1:**
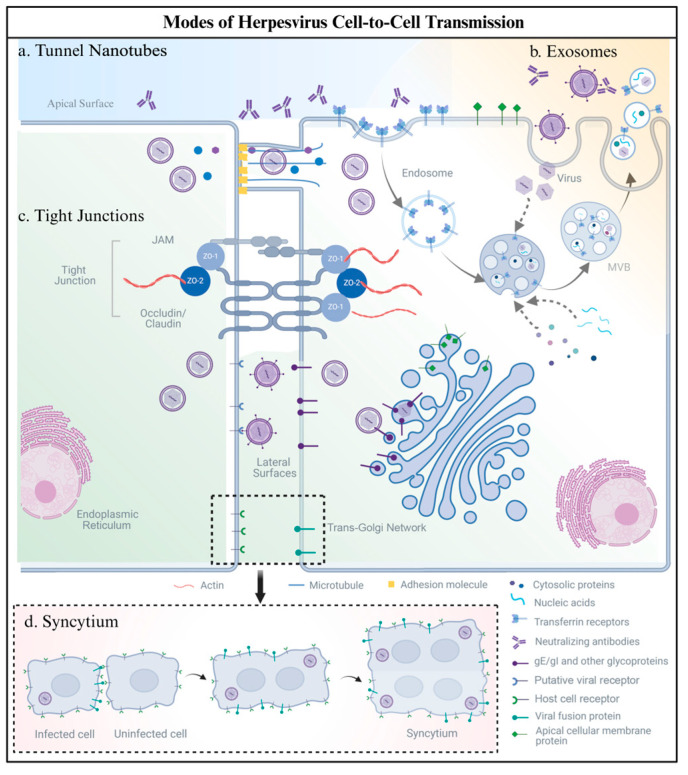
Modes of human herpesvirus cell-to-cell spread: (**a**) Tunneling Nanotubes (TNTs): Actin-/tubulin-based cytoskeletal bridges transport virions, viral RNAs, and organelles (e.g., mitochondria) between cells, serving as immune-evading conduits to bypass neutralizing antibodies. (**b**) Exosomes: Human herpesviruses hijack host ESCRT complexes and Rab GTPases. Viral components (e.g., miRNAs, capsids, or virions) are packaged into exosomes, which transfer cargo via membrane fusion or receptor uptake, evading antibody detection. (**c**) Tight Junctions (TJs): Viral glycoproteins (e.g., gE/gI) exploit polarized epithelial barriers by directing trans-Golgi network (TGN)-mediated virion trafficking to TJs, enabling basolateral spread without compromising epithelial integrity. (**d**) Syncytium: Virus-induced membrane fusion creates multinucleated cell clusters, mediated by glycoproteins (e.g., gB/gH/gL), enabling direct cytoplasmic exchange of viral particles and genomes while evading immune system surveillance. Created with BioRender.com (accessed on 11 March 2025).

Human herpesviruses (members of the family *Orthoherpesviridae*) employ diverse cell-to-cell transmission routes, including syncytium formation, tight junction (TJ) exploitation, exosomal transfer, and tunneling nanotube (TNT)-mediated spread. These processes involve intricate interactions between viral glycoproteins and host cell receptors. The main mechanisms that are used by human herpesvirus to spread from cell to cell are summarized in Figure 1.

### 2.1. Tunnel Nanotubes

Tunneling nanotubes (TNTs), recently identified as nanoscale membrane conduits (50–200 nm diameter), enable the direct intercellular transfer of organelles, proteins, and pathogens [55,56,57]. Their formation relies on dynamic cytoskeletal reorganization, particularly actin filament and microtubule remodeling, initiated by cellular signals such as growth factors, adhesion molecules (e.g., E-cadherin, β-catenin), and pathogen-derived cues that activate intracellular pathways like Rho GTPase signaling (Figure 1a) [4,58]. Human herpesviruses (members of the family *Orthoherpesviridae*) exploit TNTs as a stealth route for efficient cell-to-cell transmission, evading neutralizing antibodies and immune surveillance [57].

Human alphaherpesviruses, including HSV-1, HSV-2, and VZV, exploit TNTs as an efficient cell-to-cell transmission route to evade host immune responses. A growing body of research highlights the critical role of US3 in TNT-mediated cell-to-cell transmission. As a conserved serine/threonine kinase in human alphaherpesviruses, US3 functions as a key virulence factor, orchestrating multiple biological processes including cytoskeletal reorganization, viral replication and dissemination, apoptosis modulation, and interferon signaling pathway evasion. The viral US3 kinase orchestrates TNT formation by facilitating virion transport through TNTs via exocytosis-like processes involving viral envelope glycoproteins (gB, gD, gE) and vesicle-encapsulated particles [7]. Experimental evidence demonstrates that HSV-1 infection induces TNT formation in human corneal epithelial cells (HCECs) and Vero cells. The Arp2/3 complex (actin-related protein 2/3 complex, a key nucleator of branched actin networks) promotes TNT-mediated intercellular spread, a process effectively blocked by the inhibitor CK666, which disrupts actin polymerization [7]. Immunofluorescence and electron microscopy studies reveal HSV-1 virions and envelope glycoproteins (gD, gE) within TNTs, suggesting a vesicle-encapsulated transport mechanism [7], while HSV-2 US3 similarly induces actin-driven protrusions [8]. These findings highlight the evolutionary conservation of TNT exploitation mechanisms within the Alphaherpesvirinae subfamily. In human gammaherpesviruses, EBV utilizes glycoproteins BMRF2 and BDLF2 to induce actin-driven lamellipodia formation, enhancing viral spread through cytoskeletal remodeling contrast [14]. Beyond human herpesviruses, TNT-mediated spread is evolutionarily conserved in other members of the Alphaherpesvirinae subfamily. For instance, Pseudorabies virus (PRV) stabilizes TNTs through E-cadherin/β-catenin adhesion and acetylated microtubules [3,4], while Bovine herpesvirus 1 (BoHV-1) employs US3-induced projections to achieve antibody-resistant intercellular spread [5,6]. These findings underscore the conserved exploitation of TNTs within Alphaherpesvirinae. In contrast, the Gammaherpesvirinae subfamily exhibits divergent strategies: Murine herpesvirus 68 (MHV-68) promotes TNT-mediated dissemination via the gp48/ORF58 protein complex, highlighting lineage-specific adaptations in viral spread mechanisms [15].

Critical unresolved questions center on TNT structural plasticity (open vs. closed termini), spatiotemporal regulation by viral and host factors, and cargo transport mechanisms. For example, whether viral components traverse TNTs via free diffusion or gB-dependent vesicular transport remains debated. Furthermore, the interplay between viral triggers (e.g., US3, gp48) and cellular stress signals (e.g., inflammation, metabolic dysregulation) in modulating TNT dynamics requires systematic exploration. Emerging methodologies such as super-resolution microscopy and live-cell tracking are poised to resolve these ambiguities, while therapeutic strategies targeting cytoskeletal regulators (e.g., CK666-mediated Arp2/3 inhibition for HSV-1) or TNT-stabilizing kinases (e.g., PAK1/2) show promise in blocking this immune-evasion pathway. Elucidating TNTs’ roles in viral latency, reactivation, and tissue invasion will illuminate novel targets for disrupting herpesvirus persistence and pathogenesis.

### 2.2. Exosomes

Exosomes, nanoscale extracellular vesicles (30–150 nm) secreted by cells, serve as critical mediators of intercellular communication and immune modulation by transporting bioactive cargo (proteins, lipids, RNAs). Human herpesviruses exploit these vesicles to evade immune surveillance and enhance intercellular spread, with distinct subfamily-specific strategies (Figure 1b).

Human alphaherpesviruses such as HSV-1 exploits exosomal pathways through Rab27a GTPase-dependent mechanisms, enhancing exosome secretion to deliver viral proteins (gB, VP16, ICP5), nucleic acids, and host factors like STING protein to neighboring cells [17,18,19]. These modified exosomes facilitate immune evasion by suppressing secondary infections through host-derived proteins (e.g., Sp100A) while simultaneously promoting viral mRNA/miRNA transfer for transcellular spread [20]. Additionally, HSV also utilizes specialized vesicles like migrasomes for direct virion transfer or phagocytosis-mediated uptake, bypassing extracellular neutralization [21]. Human betaherpesviruses like HCMV reprogram host exosomal miRNA profiles by incorporating viral miRNAs, thereby subverting immune responses and facilitating spread. HCMV upregulates ESCRT (endosomal sorting complex required for transport) proteins to enhance exosome biogenesis [22]. Gammaherpesviruses such as EBV employ exosomes to deliver viral RNAs (e.g., EBERs) and miRNAs (e.g., miR-BART3, miR-BHRF1-1) to dendritic cells (DCs), inducing interferon secretion and modulating immune responses, thereby demonstrating a dual role in immune regulation and viral persistence [24,25]. Despite these advances, several unresolved questions persist: (1) the molecular basis for the selective packaging of viral components into exosomes; (2) the mechanisms governing exosome–target cell interactions (e.g., receptor-mediated docking vs. membrane fusion); and (3) the interplay between exosomal transmission and the dynamics of viral latency and reactivation. Future directions demand multidisciplinary approaches: cryo-EM and live tracking to map spatiotemporal exosomal trafficking; the mechanistic dissection of viral hijacking of host vesicular systems (e.g., Rab protein networks, ESCRT complexes); and the therapeutic development of ESCRT/Rab27a inhibitors or engineered exosomes for antiviral drug delivery. Furthermore, elucidating the synergy between exosomes and alternative routes like tunneling nanotubes (TNTs) will unravel the global regulatory network of herpesvirus dissemination, paving the way for novel strategies to disrupt viral persistence and pathogenesis [59,60].

### 2.3. Tight Junctions

Tight junctions (TJs) are critical intercellular structures in epithelial tissues, forming apical–basolateral polarity and establishing barriers against external stimuli. TJs are primarily composed of integral membrane proteins (occludin [OCLN], claudins [CLDN], junctional adhesion molecules [JAMs]) and zonula occludens (ZO)-1, -2, and -3 (Figure 1c). These junctional complexes play a pivotal role in herpesvirus dissemination, as viral entry and cell-to-cell spread often occur through either the apical or basolateral surfaces of polarized epithelial cells. During infection, the human herpesviruses spread to adjacent cells through special sites at adjacent intercellular junctions [61].

While alphaherpesviruses have been extensively studied for their TJ-mediated transmission mechanisms, betaherpesvirus and the gammaherpesvirus TJ-mediated dissemination mechanisms remain poorly characterized. In human alphaherpesviruses, newly assembled virions are trafficked specifically to cellular junctions via the trans-Golgi network (TGN) [26], with the gE/gI heterodimer facilitating basolateral targeting in polarized cells such as keratinocytes, epithelial cells, and neurons (Figure 1c) [27,28]. Mechanistically, gE/gI collaborates with tegument proteins UL7 and UL51 to stabilize membrane adhesions at junctions. Importantly, UL7’s 167–244 amino acid domain and UL51 itself are indispensable for gE localization and syncytium formation [29,30,31]. Furthermore, gE forms multicomponent complexes with VP22, gM, and ICP0 to regulate Golgi localization and secondary envelopment, with deletions in gE or gM severely impairing virion maturation and plaque formation [32,33,34]. Recent CRISPR/Cas9-based studies highlight additional viral factors: US7 enhances cell-to-cell transmission while UL56 modulates immune evasion by suppressing plaque formation [35]. Host factors, including nectin-1 and nectin-2 surface receptors, further potentiate this transmission route [36]. In contrast to the alphaherpesviruses, the gammaherpesviruses like KSHV and EBV employ distinct TJ-associated strategies: their gH/gL heterodimers bind the EphA2 receptor via conserved structural motifs, with KSHV exhibiting 236-fold higher affinity (17.5 nM vs. EBV’s 4.12 μM) due to broader interaction surfaces in gL’s N-terminal loop and L-loop2 domains. Beyond human herpesviruses, TJ exploitation strategies are evolutionarily conserved across the *Orthoherpesviridae* family. For instance, other Gammaherpesvirinae members such as Alcelaphine herpesvirus 1 (AIHV-1) and Equid herpesvirus 2 (EHV-2) similarly target EphA2 receptor-mediated pathways for junctional spread, while Murid herpesvirus 4 (MuHV-4) utilizes analogous glycoprotein–receptor interactions, suggesting potential zoonotic adaptations in viral dissemination [40]. Future studies should prioritize the mechanistic dissection of viral glycoprotein–host receptor interactions, the spatiotemporal regulation of TJ trafficking, and the therapeutic targeting of junctional complexes to disrupt viral spread.

### 2.4. Syncytium

Syncytium formation, a virus-mediated cell-to-cell transmission strategy where infected cells fuse with adjacent uninfected cells, creating multinucleated giant cells, enhances viral spread efficiency through direct cytoplasmic exchange while evading extracellular immune detection. This mechanism enhances viral spread efficiency by enabling direct cytoplasmic exchange while evading extracellular immune detection. Syncytium formation is well characterized in the human alphaherpesviruses [43,44] and the gammaherpesviruses, serving both as a pathogenic mechanism and a diagnostic marker [62,63]. Specifically, syncytia induced by HSV-1 and VZV are detectable in differentiated keratinocytes using optimized Tzanck smear techniques [64]. In contrast, betaherpesvirus-mediated syncytium formation remains poorly understood, though preliminary studies suggest HCMV exhibits cell type-specific syncytium induction [65,66,67]. Notably, recent evidence indicates this viral capability may depend on both viral glycoprotein composition and host cell tropism [68,69].

The molecular machinery of herpesvirus-induced syncytium formation involves coordinated interactions between viral glycoproteins and host factors (Figure 1d). In the human alphaherpesviruses, four core glycoproteins (gB, gD, gH/gL) have been shown to play an important role in syncytia formation [41,42]. Studies of distinct syncytial variants (e.g., gBsyn, gKsyn, UL20syn, UL24syn) reveal significant differences in the dependency on gE, gI, and UL16 across these mutants. Minor mutations in four core viral proteins (gB, gK, UL20, and UL24) can dysregulate the fusion machinery, leading to aberrant syncytium formation. The deletion of auxiliary proteins such as gE, gI, or UL16 blocks cell-to-cell transmission, forcing reliance on extracellular virion release [43]. Notably, VZV exhibits unique fusion strategies: Early studies identified the absence of a gD homolog within the Us genomic fragment, eliminating gD’s role in VZV-mediated fusion [44]. Transient expression systems demonstrate that VZV requires only gH and gL for cell–cell fusion and syncytium formation. Furthermore, VZV has evolved enhanced fusogenicity through synergistic gE-gB co-expression, which amplifies gB’s fusion activity. Quantitative analyses show comparable syncytium formation efficiency between gE-gB and gH-gL combinations, with >80% monolayer syncytium formation observed in both scenarios [44]. Intriguingly, glyproteins like gJ enhance syncytium formation despite being non-essential for replication, suggesting a “fine-tuning” mechanism for tissue-specific spread [45]. Conversely, UL24, gK, and UL20 act as fusion suppressors, potentially balancing viral dissemination with immune evasion [46]. Host factors, including PTP1B (protein tyrosine phosphatase 1B) [47], HVEM (herpesvirus entry mediator) [42], and HSPG (syndecan-1 heparan sulfate proteoglycanssyndecan-1) [48] directly modulate fusion by interacting with viral glycoproteins. This host–pathogen interplay creates a regulatory network governing syncytium development.

The betaherpesviruses, such as HCMV, demonstrate critical regulatory roles in viral infectivity and membrane fusion capacity through their glycoprotein complexes [52,53]. Emerging evidence highlights the functional divergence of the betaherpesvirus fusion machinery, where the gH/gL complex coordinates with cell-type-specific accessory proteins (e.g., UL128L) to regulate syncytium formation. For instance, the HCMV strain VR1814 induces prominent syncytia in epithelial cells and macrophages through synergistic actions of the gH/gL complex, gB, and UL128L [53]. These findings advance our understanding of mechanistic divergences in cell fusion processes across herpesvirus subfamilies. Similarly, the gammaherpesviruses members utilize immediate-early gene products to induce syncytium formation in Raji cells, highlighting their distinct regulatory pathways in membrane remodeling [16,70]. Despite significant progress in characterizing herpesvirus-induced syncytia, critical knowledge gaps persist regarding the structural basis of viral glycoprotein–host receptor interactions that orchestrate membrane fusion, as well as the conserved versus divergent molecular pathways governing this process.

## 3. Biological Advantages of Herpesvirus Cell-to-Cell Transmission

The human herpesviruses (members of the family *Orthoherpesviridae*) exploit multiple intercellular transmission mechanisms, including syncytium formation, tight junction exploitation, exosomal transfer, and tunnel nanotube (TNT) utilization. These strategies enhance viral spread efficiency while evading extracellular immune surveillance, significantly enhancing their infection and pathogenic ability.

### 3.1. Facilitating Viral Latency and Reactivation

The latency–reactivation cycle is central to herpesvirus pathogenesis. Cell-to-cell transmission mechanisms critically sustain viral genome persistence while enabling stress-triggered dissemination across host tissues. In human alphaherpesviruses (e.g., HSV-1), latent infection is sustained in sensory ganglia through immune-evading strategies such as syncytium- or exosome-mediated spread. During latency, HSV-1 utilizes host transmembrane adhesion proteins to traverse TJs between cells. This mechanism facilitates epithelial cell infection and subsequent neuronal retrograde transport, enabling the establishment of latent reservoirs in trigeminal ganglia. Such reservoirs carry a risk of progression to encephalitis [71]. Critically, this intercellular spread depends on viral glycoproteins (gB, gD, gH/gL) and gK [72]. Recent studies have shown that neurotropic alphaherpesviruses assimilate the cellular kinesin-1 motor protein during replication in epithelial cells, facilitated by the viral protein pUL36. This assimilation results in kinesin-1 becoming a structural component of the virion, which in turn facilitates the efficient transport of viral particles to neuronal nuclei, thereby enhancing viral latency and reactivation [73]. Reactivation, triggered by environmental stimuli, enables the anterograde axonal transport of virions to epithelial tissues via microtubule-associated motor proteins [74,75]. In human gammaherpesviruses (e.g., EBV), exosomes deliver viral miRNAs (miR-BARTs, miR-BHRF1) to uninfected cells. These miRNAs downregulate host anti-inflammatory miRNAs (e.g., let-7, miR-200 family), elevating inflammatory signaling and fostering oncogenesis. This exosome-mediated mechanism not only sustains viral latency but also drives tumorigenesis in EBV-associated malignancies [76,77]. These interlinked mechanisms underscore how human herpesviruses co-opt cell-to-cell transmission to choreograph latency–reactivation dynamics, offering therapeutic targets such as TNT disruptors and inhibition of the exosome formation to intercept viral persistence cycles.

### 3.2. Enhanced Infection Efficiency and Immune Evasion

Cell-to-cell transmission represents a critical strategy for human herpesviruses to enhance infection efficiency. This mechanism enables direct viral spread to adjacent cells through receptor-independent entry, bypassing extracellular immune surveillance and neutralizing antibody exposure [72]. Across all subfamilies, structural conduits like syncytia [78,79] and TNTs [4,80] shield virions during transfer. In human alphaherpesviruses (e.g., HSV-1), the US3 protein kinase promotes actin- and microtubule-containing cellular protrusions to facilitate intercellular spread and boost infection efficiency [80], while exosomal pathways encapsulate intact virions within lipid bilayers to evade immune recognition. These exosomes deliver viral miRNAs (e.g., miR-H1) that suppress type I interferon production by targeting key pathway components like STING [17,81]. Additionally, HSV-1 redirects MHC-II molecules (e.g., HLA-DR) to exosomes, reducing surface antigen presentation and evading CD4^+^ T cell detection [81]. HSV-1 also produces L-particles—enveloped secretory vesicles (exosomes)—that transfer viral proteins and cytokines to downregulate CD83 on mature dendritic cells, impairing T cell activation and enhancing immune evasion [82,83]. These strategies work synergistically to enable HSV-1 to achieve covert transmission, improve infection efficiency, and achieve persistent latency through evasion of immune surveillance. Meanwhile, in human gammaherpesviruses (e.g., EBV), infected cells undergo cell-to-cell transmission through the release of exosomes, which deliver viral glycoproteins (e.g., gB) and other immunoregulatory molecules, induce the expansion of regulatory T cells (Treg), and build an immunosuppressive microenvironment [84,85]. EBV also alters exosomal protein content to remodel the tumor microenvironment, enhancing viral persistence and promoting oncogenesis [86]. Moreover, the EBV viral genome can also be transferred to epithelial cells in the absence of EBV receptors by inducing syncytial formation [70], bypassing classical entry mechanisms. Collectively, these mechanisms establish an immunosuppressive microenvironment that underpins the development of EBV-associated lymphomas and epithelial carcinomas. Therapeutic strategies targeting viral immune-regulatory nodes, when combined with epigenetic activation, may overcome current treatment bottlenecks imposed by the immunosuppressive microenvironment mediated by viral cell-to-cell transmission. Herpesviruses universally utilize structures like syncytia [78,79] and TNTs [4,80] to shield virions from neutralizing antibodies and extracellular immune factors, minimizing exposure during transmission.

### 3.3. Augmented Pathogenicity and Tissue Invasion

Cell-to-cell transmission not only amplifies viral dissemination efficiency but also exacerbates clinical outcomes through augmented virulence mechanisms.

The human alphaherpesviruses exemplify this phenomenon through their neurotropic behavior and tissue invasiveness. Upon breaching the peripheral or central nervous system, these viruses utilize the synapses between neurons for cell-to-cell transmission, causing damage to the nervous system or establishing a lifelong latency in sensory ganglia [87]. During reactivation, VZV-induced syncytium formation is frequently observed in skin lesions and trigeminal ganglia during reactivation, where neuron–satellite cell fusion contributes to widespread neuronal injury, potentially underlying postherpetic neuralgia (PHN) [55]. PRV, a neurovirulent *Alphaherpesvirinae*, demonstrates how syncytium-mediated transmission drives neuropathology. PRV-induced syncytia disrupt neuronal electrophysiology by elevating AP (action potential) firing frequencies and establishing interneuronal electrical coupling. These aberrant synaptic activities are implicated in peripheral neuropathy, chronic pruritus, and nociceptive hypersensitivity [78,88]. Mechanistic studies in murine models reveal that PRV-gB engages TLR2 (Toll-like receptor 2) on neurons, potentiating viral cell-to-cell transmission. This interaction triggers proinflammatory cytokine release (e.g., granulocyte colony-stimulating factor [G-CSF] and interleukin-6 [IL-6]), driving acute neuroinflammation and occasionally culminating in inoculation site paralysis [89,90].

The human betaherpesviruses (HCMVs) enhance tissue invasiveness by upregulating endothelial adhesion molecules (ICAM-1, VCAM-1) at TJs [91]. These molecules facilitate viral attachment and entry while modulating host cell survival, proliferation, and inflammatory responses [92]. HCMV further disseminates via syncytia and exosome-mediated entry into mucosal epithelial cells and leukocytes, enabling systemic spread [93]. Critically, this cell-to-cell transmission strategy permits HCMV to infect liver sinusoidal Kupffer cells and endothelial cells, exacerbating hepatic inflammation and tissue damage [94]. Through these mechanisms, HCMV augments its infectivity while inducing host cell dysfunction, thereby disrupting tissue homeostasis and functionality. This dual strategy enables the virus to subvert tissue barriers and amplify its invasiveness to specific cells [65]. The human gammaherpesviruses (e.g., EBV, KSHV) remodel exosomal protein content to manipulate the tumor microenvironment. These alterations enhance viral persistence, promote oncogenesis, and facilitate immune evasion in EBV-associated malignancies [71].

## 4. Intervention Strategies Targeting Cell-to-Cell Transmission

### 4.1. Monoclonal Antibodies

Monoclonal antibodies (mAbs) represent a critical therapeutic approach for disrupting herpesviral cell-to-cell transmission, primarily targeting virion surface glycoproteins such as gD and gB. In EBV infection, gB is essential for membrane fusion, with mAbs 3A3 and 3A5 inhibiting this process through distinct mechanisms to block syncytium-mediated cell-to-cell transmission. mAb 3A3 disrupts gB binding to its coreceptor neuropilin-1 (NRP1), preventing attachment and fusion activation, while mAb 3A5 restricts the conformational transition of gB from prefusion to postfusion states, directly suppressing membrane fusion, thus blocking the syncytia formation. Both antibodies demonstrate potent neutralizing activity in murine models and in blocking syncytium-mediated cell-to-cell transmission, effectively protecting against EBV infection and mitigating lymphoproliferative disorders [54]. Synergistic strategies combining gp42-, gH-, and gL-specific neutralizing antibodies further enhance antiviral efficacy by cooperatively blocking receptor engagement, inducing glycoprotein conformational changes, and inhibiting syncytia, offering multi-target therapeutic potential for EBV-associated malignancies [95]. In HSV, mAbs targeting conserved epitopes in gD show that m27f binds a prefusion domain epitope (residues 292–297), completely neutralizing HSV by blocking cell-to-cell transmission and preventing lethal infection in mice, while 4A3 exhibit potent neutralization by binding conserved epitopes within the receptor-binding domain (residues 216–220), effectively limiting cell-to-cell transmission [49,50]. Despite their efficacy, the clinical translation of monoclonal antibodies faces challenges, including viral escape mutations driven by cell-to-cell transmission, immunogenicity concerns from incomplete humanization, and limited tissue penetration to viral reservoirs like sensory ganglia. To address these limitations, next-generation fully humanized mAbs (e.g., HDIT102) have been developed. When combined with existing therapies like HDIT101, these bispecific antibodies form multivalent antigen–antibody complexes that enhance neutralization breadth, reduce mutational escape, and potentially activate endogenous T cell responses, enhance the elimination of syncytia and other abnormal cells formed via cell-to-cell transmission [51]. Structural innovations such as single-chain variable fragments (scFvs) and single-domain antibodies (sdAbs) are being engineered to improve tissue penetration while maintaining target specificity [96,97]. Emerging delivery platforms, including antibody-drug conjugates (ADCs), may further enhance therapeutic efficacy by coupling viral neutralization with targeted cytotoxic payload delivery.

### 4.2. Antiviral Compounds

Current antiviral drug development focuses on two complementary strategies: designing novel compounds to directly inhibit viral replication and specifically targeting cell-to-cell transmission mechanisms. Recent studies have focused on host factor-targeted therapies. These studies provide new drug candidates for the treatment of human herpesviruses. For example, PTP1B inhibitors block tyrosine phosphatase activity, reducing HSV-1 syncytium formation by more than 60% and significantly inhibiting cell-to-cell transmission [47]. Prohibitin-1 (PHB1) inhibitors (e.g., RocA) interfere with the gE-PHB1 interaction, hindering viral transport via exosomes and reducing the efficiency of enveloped virus particle release [10]. These advancements highlight a multi-layered intervention strategy to cell-to-cell transmission, targeting not only viral life cycle processes (such as DNA replication and latent maintenance) but also host interaction networks (PTP1B, PHB1), laying a foundation for the development of highly effective and safe anti-herpesviruses drugs.

Additionally, certain drugs and inhibitors specifically target TJs. The BBI (Bowman–Birk inhibitor), a protease inhibitor derived from soybeans, has been reported to upregulate the expression of ZO-1, OCLN, and CLDN5 by inhibiting the ubiquitin–proteasome system in End1/E6E7 cells and reducing HSV-2-mediated ubiquitin protein degradation [37]. Some treatments, such as IL-22 therapy, can also upregulate ZO-1 and OCLN expression in HSV-2-infected cells. These regulatory mechanisms can counteract viral transmission through TJs, demonstrating the promising potential of these compounds as antiviral agents [38].

Additionally, a novel formulation, GS-1—composed of undecylenic acid and L-arginine—exerts potent antiviral activity against VZV and HSV through dual mechanisms: (1) the direct encapsulation of viral particles to prevent host cell entry; (2) the inhibition of early-stage viral replication. This unique mode of action, distinct from conventional antivirals, may suppress cell-to-cell transmission and reduce disease transmissibility [98]. Interestingly, Nelfinavir (NFV), a protease inhibitor used for HIV treatment, has provided a new approach for antiviral therapy against HSV. NFV interferes with the maturation and release process of the virus, preventing secondary envelopment of the HSV capsid. Nevertheless, the exact mechanism of this inhibitory action remains unclear, although it has been speculated that NFV may disrupt intracellular membrane sorting and trafficking, thereby inhibiting cell-to-cell transmission through TJs [39].

### 4.3. Vaccines

Vaccines capable of mediating effective immune responses play a crucial role in inhibiting viral cell-to-cell transmission. Viruses can directly spread between cells by abnormal cellular formations like syncytia structures, thereby evading the neutralizing effect of humoral immunity. However, strong immune responses, such as antibody-dependent cellular cytotoxicity (ADCC) and the binding of neutralizing antibodies, can disrupt syncytia or prevent viral cell-to-cell transmission. This mechanism also provides insights into the development of vaccines targeting herpesviruses.

The envelope gE of VZV serves dual essential roles in viral pathogenesis, coordinating virion assembly and mediating intercellular viral spread through membrane fusion events. This functional duality establishes gE as a prime immunotherapeutic target, eliciting both humoral responses (neutralizing antibodies) and cellular immunity (CD4^+^/CD8^+^ T cell activation) [99]. Capitalizing on this mechanism, GSK’s Shingrix^®^, a recombinant subunit vaccine containing gE antigen adjuvanted with AS01B, demonstrates >90% efficacy in clinical trials [11]. Its superior immunogenicity in younger cohorts correlates with robust T cell-mediated immunity (VZV-CMI), effectively suppressing viral reactivation in sensory ganglia through the cytotoxic lymphocyte-mediated clearance of infected neurons [12]. These findings demonstrate that gE serves as a core immunogen, eliciting robust humoral and cellular immune responses against herpesviruses. Notably, the co-expression of gE with gI enhances antigen processing and presentation via MHC-II pathways, amplifying Th1-polarized immune responses critical for long-term immunological memory [13]. Furthermore, structural vaccinology approaches targeting the gH/gL heterodimer complex, a conserved fusogen essential for viral entry, have shown promise in generating cross-neutralizing antibodies that block cell-to-cell transmission through the steric hindrance of membrane fusion machinery, providing a new direction for VZV development [100].

EBV is a virus closely associated with various human cancers, and its latent proteins play a key role in the onset and progression of EBV-associated cancers. These latent proteins are not only persistently expressed in tumor cells but also promote viral cell-to-cell transmission, cell proliferation, survival, and immune evasion through various mechanisms, thereby driving tumorigenesis. Therefore, therapeutic vaccines targeting EBV latent proteins have become a hot research topic. Research teams are actively exploring mRNA-based EBV therapeutic vaccines and nanoparticle-based vaccines to achieve precise delivery and immune enhancement. Parallel efforts by academic teams, such as Prof. Zeng Musheng’s group at Sun Yat-sen University, have engineered truncated EBV latency antigens (e.g., EBNA1ΔGA92-327) to enhance MHC-I presentation, achieving significant tumor regression in murine models through potent CD8^+^ T cell activation, thus reducing cell-to-cell transmission [101]. Dr. Liu Lixin’s team at Sun Yat-sen University developed a novel EBV subunit nanoparticle vaccine in combination with the immune checkpoint inhibitor anti-PD-L1. This combination not only triggered immune responses via the vaccine but also counteracted immune tolerance that leads to tumor progression. This vaccine may indirectly suppress viral cell-to-cell transmission by activating the immune system to recognize and attack EBV-infected cells [102]. Furthermore, Dr. Zhang Xiao and colleagues at Sun Yat-sen University formulated three nanoparticle vaccines by co-delivering molecular adjuvants (CpG and MPLA) and antigens (gHgL, gB, or gp42). These nanoparticle vaccines effectively activated dendritic cells and germinal center responses, inducing strong humoral and cellular immune responses. They displayed potent immunogenicity and effectively recognized abnormal cells involved in viral cell-to-cell transmission, such as syncytia. This suggests that the vaccine enhances the immune system’s ability to identify and clear abnormal cells associated with viral transmission, thereby inhibiting viral cell-to-cell spread [103]. EBV vaccine development has shifted from a single preventive strategy to diversified therapeutic and combination interventions, with significant breakthroughs driven by mRNA and nanotechnology. Currently, WGc-043, the world’s first mRNA therapeutic vaccine targeting EBV-specific antigens, has received U.S. FDA approval for clinical trials. Utilizing a proprietary lipid nanoparticle (LNP) delivery system and engineered immune enhancers, this vaccine activates cytotoxic T cell and antibody responses to inhibit viral cell-to-cell transmission, offering a potent and safe innovative therapy for EBV-associated malignancies such as nasopharyngeal carcinoma [16].

In parallel, multiple HCMV vaccine candidates targeting viral entry machinery are undergoing clinical evaluation, with a predominant focus on the trimeric (gH/gL/gO) and pentameric (gH/gL/UL128/130/131A) glycoprotein complexes that mediate critical host–cell entry processes [104]. Leading strategies in prophase focus on the trimeric gH/gL/gO complex. Preclinical studies demonstrate that trimeric glycoprotein complexes elicit potent neutralizing antibodies capable of countering HCMV immune evasion mechanisms, positioning this antigenic configuration as a promising candidate for next-generation vaccine development [105]. A key advancement is the mRNA-1647 candidate currently in Phase III trials (NCT05085366), which combines six mRNA components: one encoding gB and five encoding pentamer subunits [23]. This candidate vaccine can induce effective and broad neutralizing activity, as well as enhanced antibody-dependent cellular cytotoxicity (ADCC), thereby inhibiting viruses transmitted via cell-to-cell transmission and enhancing the protective efficacy of the vaccine [106]. Notably, pentamer-specific neutralizing antibodies are prevalent in sera from transplant recipients and pregnant individuals, demonstrating potent inhibition of both cell-free and cell-associated viral spread—a dual mechanism critical for preventing congenital transmission and post-transplant reactivation [104]. HCMV vaccine development has entered a pivotal stage, with the integration of trimeric/pentameric glycoprotein targets and mRNA technology emerging as the cornerstone of breakthrough strategies. To achieve broad-spectrum protection, future efforts must address persistent challenges, including viral genetic diversity, immune evasion tactics, and the need to elicit robust cellular immunity. Cross-disciplinary innovations such as structural vaccinology-guided antigen design and epitope optimization are now being leveraged to overcome these barriers.

The development of HSV vaccines remains confined to clinical trial phases, challenged by the complex genetic structure and cell-to-cell transmission process of HSV, the reactivation of latent infection, and a variety of glycoproteins (gE, gB, gD, gH/gL). These factors collectively impede the induction of durable immune protection across diverse populations, with no FDA-approved vaccine to date [107,108]. The development of HSV vaccines faces serotype-specific efficacy challenges. GSK’s gD subunit vaccine demonstrated partial protection in HSV-1/HSV-2 seronegative women but showed limited efficacy in broader populations, including those with pre-existing antibodies. This limited efficacy may be attributed to the virus’s ability to spread via cell-to-cell transmission, which undermines the vaccine’s immunoprotective effects, underscoring the need for cross-reactive immunization strategies. In contrast, the success of Shingrix^®^ (GSK’s VZV) highlights critical lessons for herpesvirus vaccine design: high-affinity antibodies must synergize with robust cellular immunity to suppress intercellular viral spread, and targeting conserved entry/fusion machinery may overcome immune evasion. While HSV-neutralizing antibodies exhibit a partial inhibition of free virions, they frequently fail to block cell-to-cell transmission, a key bottleneck in clinical protection [109]. Emerging multi-target strategies that combine antibodies or multivalent vaccine components demonstrate enhanced protective efficacy by simultaneously engaging distinct viral antigens. In HSV vaccine development, the fully humanized monoclonal antibodies HDIT101 and HDIT102 exhibit synergistic potential in clinical trials, effectively suppressing viral recurrence through integrated mechanisms: blocking cell-to-cell transmission, enhancing Fc-mediated phagocytic clearance, and stimulating T cell-mediated adaptive immunity [51].

## 5. Summary and Perspectives

Members of the family *Orthoherpesviridae* represent ubiquitous human pathogens that employ sophisticated cell-to-cell transmission strategies to enhance infectivity, evade immune detection, augment pathogenicity, and establish persistent latent infections. While significant advances have been made in understanding herpesvirus cell-to-cell spread, critical knowledge gaps remain in mechanistic characterization and therapeutic targeting.

Future investigations should adopt systematic approaches to address three fundamental questions: the divergent transmission mechanisms among alphaherpesviruses (e.g., HSV-1), betaherpesviruses (e.g., HCMV), and gammaherpesviruses (e.g., KSHV). The dynamic interplay between viral glycoproteins and host cell receptors during transmission and molecular interactions governs viral spread at nanometer resolution. Advanced imaging modalities, including super-resolution fluorescence microscopy and cryo-electron tomography, enable the real-time tracking of viral–host protein interactions in live cells, yielding high-resolution spatial-temporal data to map molecular-scale structural rearrangements during transmission [110]. Complementary biochemical approaches such as AP-MS (affinity purification–mass spectrometry) and crosslinking proteomics systematically identify and functionally characterize key viral and host proteins orchestrating cell-to-cell spread, providing mechanistic insights into transmission pathways [110]. Advanced genetic tools such as CRISPR/Cas9-mediated genome editing and BAC (bacterial artificial chromosome) recombineering permit the precise manipulation of viral genomes to establish isogenic mutant strains for comparative transmission studies [111], through cell biology and molecular biology methods to compare different virus strains in the process of transmission between cells, to clarify the key molecules and signal pathways.

The cell-to-cell transmission strategies of herpesviruses not only govern their own infection dynamics but also shape the coinfection microenvironment through unique molecular mechanisms, leading to complex viral interactions. Studies reveal that HSV-1 can reactivate latent EBV into lytic cycles by hijacking host signaling pathways (e.g., CREB activation), where the viral US3 kinase directly phosphorylates CREB to upregulate the EBV BZLF1 promoter activity [112]. This illustrates a synergistic mechanism whereby herpesviruses co-opt host transcriptional networks to facilitate secondary viral replication. Such regulation may involve the intercellular transfer of viral components (e.g., US3) or inflammatory mediators (e.g., TNF-α), the latter being pivotal in HSV-HIV coinfection: HSV-1 enhances HIV infectivity in T cells by upregulating CCR5 expression on Langerhans cells (LCs) [113]. Notably, HSV-1’s “helper” role in AAV replication operates through a distinct dimension—its ICP8 protein alters AAV genome end recombination patterns [114,115], mirroring HSV’s intrinsic genome recombination machinery. Collectively, these findings demonstrate that herpesvirus-driven cell-to-cell transmission establishes both physical microenvironments (e.g., syncytium formation, TNTs) and molecular networks (kinase activation, cytokine release), which can either synergistically enhance viral dissemination or selectively interfere with competitor replication. The bidirectional outcomes depend on the molecular characteristics of co-infecting viruses and the host cell state.

Despite advancements in therapeutic strategies targeting cell-to-cell transmission, critical barriers hinder clinical translation, including unresolved challenges in drug pharmacokinetic optimization, off-target toxicity mitigation, immune evasion circumvention, and latent reservoir reactivation control. A paramount challenge lies in balancing immunogenic efficacy with safety profiles while stimulating robust cellular immunity to suppress viral spread. To address these hurdles, next-generation interventions should prioritize the structure-guided development of monoclonal antibodies (e.g., anti-gD/gB) and small-molecule inhibitors (e.g., US3 kinase antagonists) targeting conserved glycoprotein epitopes essential for cell-to-cell transmission. The concurrent development of multi-target combination therapies (e.g., the simultaneous blockade of TNT formation and exosome secretion) could counter viral immune evasion. Concurrently, integrating multidisciplinary approaches spanning virology, structural biology, and computational immunology is imperative to bridge mechanistic insights with clinical implementation. Such synergies will accelerate the translation of fundamental discoveries into precision therapies capable of disrupting viral dissemination pathways and improving clinical management of herpesvirus-associated pathologies.

Notably, comparative virology studies of the family *Alloherpesviridae* provide evolutionary insights into herpesvirus transmission mechanisms. CyHV-3 (Cyprinid herpesvirus 3) exemplifies temperature-dependent transmission strategies in fish hosts, where histological analysis reveals gill epithelial necrosis and renal tubular damage mediated by syncytium formation. The observed temperature-dependent transmission mechanism in *alloherpesvirus* transmission provides a conceptual framework for exploring whether human herpesviruses (e.g., VZV, EBV) could potentially co-opt host metabolic changes such as fever-induced TNT upregulation as a hypothetical transmission-enhancing strategy [116].

In conclusion, deciphering the molecular determinants of herpesviruses’ cell-to-cell transmission represents both a fundamental virological challenge and a therapeutic imperative. Continued investigation using next-generation technologies will advance our capacity to disrupt viral spread mechanisms and develop effective countermeasures against herpesvirus-associated pathologies.

## Figures and Tables

**Table 1:** Classification of human herpesviruses in the family *Orthoherpesviridae*.

Subfamily	Genus	Human Virus Species	Virus Name	Abbreviation
*Alphaherpesvirinae*	*Simplexvirus*	*Simplexvirus humanalpha1*	*human alphaherpesvirus 1 (Herpes simplex virus type 1)*	HSV-1
		*Simplexvirus humanalpha2*	*human alphaherpesvirus 2 (Herpes simplex virus type 2)*	HSV-2
	*Varicellovirus*	*Varicellovirus humanalpha3*	*human alphaherpesvirus 3* (*varicella-zoster virus*)	*VZV*
*Betaherpesvirinae*	*Cytomegalovirus*	*Cytomegalovirus humanbeta5*	*human betaherpesvirus 5 (human cytomegalovirus)*	*HCMV*
	*Roseolovirus*	*Roseolovirus humanbeta6a*	*human betaherpesvirus 6A (human herpesvirus 6A)*	*HHV6A*
		*Roseolovirus humanbeta6b*	*human betaherpesvirus 6B (human herpesvirus 6B)*	*HHV6B*
		*Roseolovirus humanbeta7*	*human betaherpesvirus7 (human herpesvirus 7)*	*HHV7*
*Gammaherpesvirinae*	*Lymphocryptovirus*	*Lymphocryptovirus humangamma4*	*human gammaherpesvirus 4 (Epstein–Barr virus)*	*EBV*
	*Rhadinovirus*	*Rhadinovirus humangamma8*	*human gammaherpesvirus 8 (Kaposi’s sarcoma-associated herpesvirus)*	*KSHV*

The classification of human herpesviruses presented in this table follows the International Committee on the Taxonomy of Viruses (ICTV) Master Species List (MSL) as of May 2023. The taxonomy is restricted to human-associated viruses within the family *Orthoherpesviridae*. For complete ICTV classification criteria and updates, refer to the official ICTV repository.

**Table 2:** Cell-to-cell transmission, glycoproteins, and interventions in human herpesviruses.

Transmission Mode	Subfamily	Key Glycoproteins	Host Receptors	Intervention Strategies (Include Under Development)	Intervention Targets	Refs.
**Tunneling Nanotubes (TNTs)**	*Alphaherpesvirinae* (e.g., HSV-1)	gB, gD, gE, US3 kinase	Actin cytoskeleton (Arp2/3 complex), E-cadherin/β-catenin	CK666 inhibitors, PHB1 inhibitors (RocA), Shingrix^®^ vaccine (GlaxoSmithKline, Brentford, UK)	PAK1/2, PHB1, gE, gE/gI	[3,4,5,6,7,8,9,10,11,12,13]
	*Gammaherpesvirinae* (e.g., EBV)	BMRF2, BDLF2, gp48/ORF58	Not explicitly mentioned	EBV mRNA vaccine (WGc-043), liposomal nanovaccinations	gHgL, gB, gp42	[14,15,16]
**Exosomes**	*Alphaherpesvirinae* (e.g., HSV-1)	gB, VP16, ICP5, miRNAs	Rab27a, STING, Sp100A	Shingrix^®^ vaccine (VZV)	gE/gI	[11,12,13,17,18,19,20,21]
	*Betaherpesvirina*e (e.g., HCMV)	gB, UL128L, miRNAs,	ESCRT proteins	HCMV mRNA (mRNA-1647)	gB and pentameric subunits (gH/gL/UL128/130/131A)	[22,23]
	*Gammaherpesvirinae* (e.g., EBV)	EBERs, miR-BART3, miR-BHRF1-1	DCs, IFN	EBV mRNA vaccine (WGc-043), liposomal nanovaccinations	gHgL, gB, gp42	[16,24,25]
**Tight Junctions (TJs)**	*Alphaherpesvirinae* (e.g., HSV-1,VZV)	gE, gI, gM, UL7, UL51, VP22, UL56, US7, ICP0	TGN, Nectin-1, Nectin-2	PHB1) inhibitors (RocA), BBI inhibitors, IL-22 therapy, Nelfinavir (NFV), Shingrix^®^ vaccine (VZV)	End1/E6E7, ZO-1, OCLN, TGN, gE/gI	[10,11,12,13,26,27,28,29,30,31,32,33,34,35,36,37,38,39]
	*Gammaherpesvirinae* (e.g., EBV, KSHV)	gH/gL	EphA2 receptor	EBV mRNA vaccine (WGc-043), liposomal nanovaccinations	gHgL, gB, gp42	[16,40]
**Syncytium**	*Alphaherpesvirinae* (e.g., HSV-1, VZV)	gB, gD (VZV exclusive), gH/gL, gE, gK, gJ, UL16, UL20, UL24	PTP1B, HVEM, HSPG	PTP1B inhibitors, PHB1 inhibitors (RocA). mAb: m27f, 4A3, HDIT101, HDIT10. Shingrix^®^ vaccine (VZV)	PTP1B, gD (residues 292–297, 216–220), gB, gE/gI	[10,11,41,42,43,44,45,46,47,48,49,50,51]
	*Betaherpesvirinae* (e.g., HCMV)	gH/gL, gB, UL128L	Not explicitly mentioned	HCMV mRNA (mRNA-1647)	gB and pentameric subunits (gH/gL/UL128/130/131A)	[23,52,53]
	*Gammaherpesvirinae* (e.g., EBV, KSHV)	Immediate-early gene products	Raji cells, specific receptors	mAb: 3A3, 3A5. EBV mRNA vaccine (WGc-043), liposomal nanovaccinations	gB, NRP1, gH/gL, gB, gp42	[16,24,25,54]

## References

[1] Wijesinghe V.N., Farouk I.A., Zabidi N.Z., Puniyamurti A., Choo W.S., Lal S.K. (2021). Current vaccine approaches and emerging strategies against herpes simplex virus (HSV). Expert Rev. Vaccines.

[2] Guo H., Yi R.P., Xu S.T. (2021). Progress of molecular epidemiological research on human herpes virus. Int. J. Virol..

[3] Jansens R.J.J., Tishchenko A., Favoreel H.W. (2020). Bridging the Gap: Virus Long-Distance Spread via Tunneling Nanotubes. J. Virol..

[4] Jansens R.J.J., Van den Broeck W., De Pelsmaeker S., Lamote J.A.S., Van Waesberghe C., Couck L., Favoreel H.W. (2017). Pseudorabies Virus US3-Induced Tunneling Nanotubes Contain Stabilized Microtubules, Interact with Neighboring Cells via Cadherins, and Allow Intercellular Molecular Communication. J. Virol..

[5] Ladelfa M.F., Kotsias F., Del Médico Zajac M.P., Van den Broeke C., Favoreel H., Romera S.A., Calamante G. (2011). Effect of the US3 protein of bovine herpesvirus 5 on the actin cytoskeleton and apoptosis. Vet. Microbiol..

[6] Panasiuk M., Rychłowski M., Derewońko N., Bieńkowska-Szewczyk K. (2018). Tunneling Nanotubes as a Novel Route of Cell-to-Cell Spread of Herpesviruses. J. Virol..

[7] Wang J., Shang K.T., Ma Q.H., Dong Z.Y., Chen Y.H., Yao Y.F. (2023). Herpes Simplex Virus Type 1 Infection Induces the Formation of Tunneling Nanotubes. Microorganisms.

[8] Finnen R.L., Roy B.B., Zhang H., Banfield B.W. (2010). Analysis of filamentous process induction and nuclear localization properties of the HSV-2 serine/threonine kinase Us3. Virology.

[9] Sun M., Hou L., Tang Y.D., Liu Y., Wang S., Wang J., Shen N., An T., Tian Z., Cai X. (2017). Pseudorabies virus infection inhibits autophagy in permissive cells in vitro. Sci. Rep..

[10] Watanabe M., Arii J., Takeshima K., Fukui A., Shimojima M., Kozuka-Hata H., Oyama M., Minamitani T., Yasui T., Kubota Y. (2021). Prohibitin-1 Contributes to Cell-to-Cell Transmission of Herpes Simplex Virus 1 via the MAPK/ERK Signaling Pathway. J. Virol..

[11] Huang L., Zhao T., Zhao W., Shao A., Zhao H., Ma W., Gong Y., Zeng X., Weng C., Bu L. (2024). Herpes zoster mRNA vaccine induces superior vaccine immunity over licensed vaccine in mice and rhesus macaques. Emerg. Microbes Infect..

[12] Lecrenier N., Beukelaers P., Colindres R., Curran D., De Kesel C., De Saegher J.P., Didierlaurent A.M., Ledent E.Y., Mols J.F., Mrkvan T. (2018). Development of adjuvanted recombinant zoster vaccine and its implications for shingles prevention. Expert Rev. Vaccines.

[13] Zerboni L., Sen N., Oliver S.L., Arvin A.M. (2014). Molecular mechanisms of varicella zoster virus pathogenesis. Nat. Rev. Microbiol..

[14] Loesing J.-B., Di Fiore S., Ritter K., Fischer R., Kleines M. (2009). Epstein–Barr virus BDLF2–BMRF2 complex affects cellular morphology. J. Gen. Virol..

[15] Gill M.B., Edgar R., May J.S., Stevenson P.G. (2008). A gamma-herpesvirus glycoprotein complex manipulates actin to promote viral spread. PLoS ONE.

[16] Möhl B.S., Chen J., Park S.J., Jardetzky T.S., Longnecker R. (2017). Epstein-Barr Virus Fusion with Epithelial Cells Triggered by gB Is Restricted by a gL Glycosylation Site. J. Virol..

[17] Kalamvoki M., Du T., Roizman B. (2014). Cells infected with herpes simplex virus 1 export to uninfected cells exosomes containing STING, viral mRNAs, and microRNAs. Proc. Natl. Acad. Sci. USA.

[18] Kalamvoki M., Deschamps T. (2016). Extracellular vesicles during Herpes Simplex Virus type 1 infection: An inquire. Virol. J..

[19] Deschamps T., Kalamvoki M. (2018). Extracellular Vesicles Released by Herpes Simplex Virus 1-Infected Cells Block Virus Replication in Recipient Cells in a STING-Dependent Manner. J. Virol..

[20] Ma Y., Li J., Dong H., Yang Z., Zhou L., Xu P. (2022). PML Body Component Sp100A Restricts Wild-Type Herpes Simplex Virus 1 Infection. J. Virol..

[21] Liu Y., Zhu Z., Li Y., Yang M., Hu Q. (2023). Migrasomes released by HSV-2-infected cells serve as a conveyance for virus spread. Virol. Sin..

[22] Streck N.T., Zhao Y., Sundstrom J.M., Buchkovich N.J. (2020). Human Cytomegalovirus Utilizes Extracellular Vesicles To Enhance Virus Spread. J. Virol..

[23] Panther L., Basnet S., Fierro C., Brune D., Leggett R., Peterson J., Pickrell P., Lin J., Wu K., Lee H. (2023). 2892. Safety and Immunogenicity of mRNA-1647, an mRNA-Based Cytomegalovirus Vaccine in Healthy Adults: Results of a Phase 2, Randomized, Observer-Blind, Placebo-Controlled, Dose-Finding Trial. Open Forum Infect. Dis..

[24] Ahmed W., Tariq S., Khan G. (2018). Tracking EBV-encoded RNAs (EBERs) from the nucleus to the excreted exosomes of B-lymphocytes. Sci. Rep..

[25] Xiao Z., Zhang S. (2020). Advances on the mechanism of Epstein-Barr virus transmission in vivo. J. Clin. Pathol. Res..

[26] McMillan T.N., Johnson D.C. (2001). Cytoplasmic domain of herpes simplex virus gE causes accumulation in the trans-Golgi network, a site of virus envelopment and sorting of virions to cell junctions. J. Virol..

[27] Johnson D.C., Huber M.T. (2002). Directed egress of animal viruses promotes cell-to-cell spread. J. Virol..

[28] Farnsworth A., Johnson D.C. (2006). Herpes simplex virus gE/gI must accumulate in the trans-Golgi network at early times and then redistribute to cell junctions to promote cell-cell spread. J. Virol..

[29] Roller R.J., Haugo A.C., Yang K., Baines J.D. (2014). The herpes simplex virus 1 UL51 gene product has cell type-specific functions in cell-to-cell spread. J. Virol..

[30] Roller R.J., Fetters R. (2015). The herpes simplex virus 1 UL51 protein interacts with the UL7 protein and plays a role in its recruitment into the virion. J. Virol..

[31] Feutz E., McLeland-Wieser H., Ma J., Roller R.J. (2019). Functional interactions between herpes simplex virus pUL51, pUL7 and gE reveal cell-specific mechanisms for epithelial cell-to-cell spread. Virology.

[32] Stylianou J., Maringer K., Cook R., Bernard E., Elliott G. (2009). Virion incorporation of the herpes simplex virus type 1 tegument protein VP22 occurs via glycoprotein E-specific recruitment to the late secretory pathway. J. Virol..

[33] Maringer K., Stylianou J., Elliott G. (2012). A network of protein interactions around the herpes simplex virus tegument protein VP22. J. Virol..

[34] Farnsworth A., Wisner T.W., Johnson D.C. (2007). Cytoplasmic residues of herpes simplex virus glycoprotein gE required for secondary envelopment and binding of tegument proteins VP22 and UL11 to gE and gD. J. Virol..

[35] Bzik D.J., Fox B.A., DeLuca N.A., Person S. (1984). Nucleotide sequence of a region of the herpes simplex virus type 1 gB glycoprotein gene: Mutations affecting rate of virus entry and cell fusion. Virology.

[36] Delboy M.G., Patterson J.L., Hollander A.M., Nicola A.V. (2006). Nectin-2-mediated entry of a syncytial strain of herpes simplex virus via pH-independent fusion with the plasma membrane of Chinese hamster ovary cells. Virol. J..

[37] Liu Y., Xu X.Q., Zhang B., Gu J., Meng F.Z., Liu H., Zhou L., Wang X., Hou W., Ho W.Z. (2018). Bowman-Birk Inhibitor Suppresses Herpes Simplex Virus Type 2 Infection of Human Cervical Epithelial Cells. Viruses.

[38] Xu X.Q., Liu Y., Zhang B., Liu H., Shao D.D., Liu J.B., Wang X., Zhou L.N., Hu W.H., Ho W.Z. (2019). IL-22 suppresses HSV-2 replication in human cervical epithelial cells. Cytokine.

[39] Kalu N.N., Desai P.J., Shirley C.M., Gibson W., Dennis P.A., Ambinder R.F. (2014). Nelfinavir inhibits maturation and export of herpes simplex virus 1. J. Virol..

[40] Su C., Wu L., Chai Y., Qi J., Tan S., Gao G.F., Song H., Yan J. (2020). Molecular basis of EphA2 recognition by gHgL from gammaherpesviruses. Nat. Commun..

[41] Atanasiu D., Saw W.T., Cohen G.H., Eisenberg R.J. (2010). Cascade of Events Governing Cell-Cell Fusion Induced by Herpes Simplex Virus Glycoproteins gD, gH/gL, and gB. J. Virol..

[42] Terry-Allison T., Montgomery R.I., Whitbeck J.C., Xu R., Cohen G.H., Eisenberg R.J., Spear P.G. (1998). HveA (Herpesvirus Entry Mediator A), a Coreceptor for Herpes Simplex Virus Entry, also Participates in Virus-Induced Cell Fusion. J. Virol..

[43] Carmichael J.C., Wills J.W. (2019). Differential Requirements for gE, gI, and UL16 among Herpes Simplex Virus 1 Syncytial Variants Suggest Unique Modes of Dysregulating the Mechanism of Cell-to-Cell Spread. J. Virol..

[44] Maresova L., Pasieka T.J., Grose C. (2001). Varicella-zoster Virus gB and gE coexpression, but not gB or gE alone, leads to abundant fusion and syncytium formation equivalent to those from gH and gL coexpression. J. Virol..

[45] Liu Y., Guan X., Li C., Ni F., Luo S., Wang J., Zhang D., Zhang M., Hu Q. (2018). HSV-2 glycoprotein J promotes viral protein expression and virus spread. Virology.

[46] Ruan P., Wang M., Cheng A., Zhao X., Yang Q., Wu Y., Zhang S., Tian B., Huang J., Ou X. (2023). Mechanism of herpesvirus UL24 protein regulating viral immune escape and virulence. Front. Microbiol..

[47] Carmichael J.C., Yokota H., Craven R.C., Schmitt A., Wills J.W. (2018). The HSV-1 mechanisms of cell-to-cell spread and fusion are critically dependent on host PTP1B. PLoS Pathog..

[48] Karasneh G.A., Ali M., Shukla D. (2011). An important role for syndecan-1 in herpes simplex virus type-1 induced cell-to-cell fusion and virus spread. PLoS ONE.

[49] Du R., Wang L., Xu H., Wang Z., Zhang T., Wang M., Ning Y., Deng F., Hu Z., Wang H. (2017). A novel glycoprotein D-specific monoclonal antibody neutralizes herpes simplex virus. Antivir. Res..

[50] Tian R., Ju F., Yu M., Liang Z., Xu Z., Zhao M., Qin Y., Lin Y., Huang X., Chang Y. (2022). A potent neutralizing and protective antibody against a conserved continuous epitope on HSV glycoprotein D. Antivir. Res..

[51] Seyfizadeh N., Kalbermatter D., Imhof T., Ries M., Müller C., Jenner L., Blumenschein E., Yendrzheyevskiy A., Grün F., Moog K. (2024). Development of a highly effective combination monoclonal antibody therapy against Herpes simplex virus. J. Biomed. Sci..

[52] Kinzler E.R., Compton T. (2005). Characterization of human cytomegalovirus glycoprotein-induced cell-cell fusion. J. Virol..

[53] Cimato G., Zhou X., Brune W., Frascaroli G. (2024). Human cytomegalovirus glycoprotein variants governing viral tropism and syncytium formation in epithelial cells and macrophages. J. Virol..

[54] Zhang X., Hong J., Zhong L., Wu Q., Zhang S., Zhu Q., Chen H., Wei D., Li R., Zhang W. (2022). Protective anti-gB neutralizing antibodies targeting two vulnerable sites for EBV-cell membrane fusion. Proc. Natl. Acad. Sci. USA.

[55] Rustom A., Saffrich R., Markovic I., Walther P., Gerdes H.H. (2004). Nanotubular highways for intercellular organelle transport. Science.

[56] Zurzolo C. (2021). Tunneling nanotubes: Reshaping connectivity. Curr. Opin. Cell Biol..

[57] Lv W., Li Z., Wang S., He J., Zhang L. (2024). A role for tunneling nanotubes in virus spread. Front. Microbiol..

[58] Wang X.-Q., Yu X.-W., Liu M.-Y., Guan Y.-T. (2015). Progress in promoting mechanism of tunneling nanotubes formation. Acad. J. Second. Mil. Med. Univ..

[59] Meckes D.G., Shair K.H.Y., Marquitz A.R., Kung C.-P., Edwards R.H., Raab-Traub N. (2010). Human tumor virus utilizes exosomes for intercellular communication. Proc. Natl. Acad. Sci. USA.

[60] Marsh M., Helenius A. (2006). Virus Entry: Open Sesame. Cell.

[61] Cifuentes-Munoz N., El Najjar F., Dutch R.E. (2020). Viral cell-to-cell spread: Conventional and non-conventional ways. Adv. Virus Res..

[62] Yamamoto Y., Yamamoto T., Aoyama Y., Fujimoto W. (2019). Cell-to-cell transmission of HSV-1 in differentiated keratinocytes promotes multinucleated giant cell formation. J. Dermatol. Sci..

[63] Wang W., Zheng Q., Pan D., Yu H., Fu W., Liu J., He M., Zhu R., Cai Y., Huang Y. (2020). Near-atomic cryo-electron microscopy structures of varicella-zoster virus capsids. Nat. Microbiol..

[64] Yamamoto T., Aoyama Y. (2020). Detection of multinucleated giant cells in differentiated keratinocytes with herpes simplex virus and varicella zoster virus infections by modified Tzanck smear method. J. Dermatol..

[65] Tang J., Frascaroli G., Zhou X., Knickmann J., Brune W. (2021). Cell Fusion and Syncytium Formation in Betaherpesvirus Infection. Viruses.

[66] Sinzger C., Bissinger A.L., Viebahn R., Oettle H., Radke C., Schmidt C.A., Jahn G. (1999). Hepatocytes are permissive for human cytomegalovirus infection in human liver cell culture and In vivo. J. Infect. Dis..

[67] Booth J.C., Beesley J.E., Stern H. (1978). Syncytium formation caused by human cytomegalovirus in human embryonic lung fibroblasts. Arch. Virol..

[68] Gerna G., Percivalle E., Perez L., Lanzavecchia A., Lilleri D. (2016). Monoclonal Antibodies to Different Components of the Human Cytomegalovirus (HCMV) Pentamer gH/gL/pUL128L and Trimer gH/gL/gO as well as Antibodies Elicited during Primary HCMV Infection Prevent Epithelial Cell Syncytium Formation. J. Virol..

[69] Vo M., Aguiar A., McVoy M.A., Hertel L. (2020). Cytomegalovirus Strain TB40/E Restrictions and Adaptations to Growth in ARPE-19 Epithelial Cells. Microorganisms.

[70] Bayliss G.J., Wolf H. (1981). An Epstein--Barr virus early protein induces cell fusion. Proc. Natl. Acad. Sci. USA.

[71] Bello-Morales R., López-Guerrero J.A. (2018). Extracellular Vesicles in Herpes Viral Spread and Immune Evasion. Front. Microbiol..

[72] Madavaraju K., Koganti R., Volety I., Yadavalli T., Shukla D. (2021). Herpes Simplex Virus Cell Entry Mechanisms: An Update. Front. Cell. Infect. Microbiol..

[73] Pegg C.E., Zaichick S.V., Bomba-Warczak E., Jovasevic V., Kim D., Kharkwal H., Wilson D.W., Walsh D., Sollars P.J., Pickard G.E. (2021). Herpesviruses assimilate kinesin to produce motorized viral particles. Nature.

[74] Nicoll M.P., Hann W., Shivkumar M., Harman L.E., Connor V., Coleman H.M., Proença J.T., Efstathiou S. (2016). The HSV-1 Latency-Associated Transcript Functions to Repress Latent Phase Lytic Gene Expression and Suppress Virus Reactivation from Latently Infected Neurons. PLoS Pathog..

[75] Nicoll M.P., Proença J.T., Connor V., Efstathiou S. (2012). Influence of herpes simplex virus 1 latency-associated transcripts on the establishment and maintenance of latency in the ROSA26R reporter mouse model. J. Virol..

[76] Polakovicova I., Jerez S., Wichmann I.A., Sandoval-Bórquez A., Carrasco-Véliz N., Corvalán A.H. (2018). Role of microRNAs and Exosomes in Helicobacter pylori and Epstein-Barr Virus Associated Gastric Cancers. Front. Microbiol..

[77] Wen F., Han Y., Zhang H., Zhao Z., Wang W., Chen F., Qin W., Ju J., An L., Meng Y. (2024). Epstein-Barr virus infection upregulates extracellular OLFM4 to activate YAP signaling during gastric cancer progression. Nat. Commun..

[78] Ambrosini A.E., Enquist L.W. (2015). Cell-Fusion Events Induced by α-Herpesviruses. Future Virol..

[79] Sarfo A., Starkey J., Mellinger E., Zhang D., Chadha P., Carmichael J., Wills J.W. (2017). The UL21 Tegument Protein of Herpes Simplex Virus 1 Is Differentially Required for the Syncytial Phenotype. J. Virol..

[80] Favoreel H.W., Van Minnebruggen G., Adriaensen D., Nauwynck H.J. (2005). Cytoskeletal rearrangements and cell extensions induced by the US3 kinase of an alphaherpesvirus are associated with enhanced spread. Proc. Natl. Acad. Sci. USA.

[81] Temme S., Eis-Hübinger A.M., McLellan A.D., Koch N. (2010). The herpes simplex virus-1 encoded glycoprotein B diverts HLA-DR into the exosome pathway. J. Immunol..

[82] Heilingloh C.S., Kummer M., Mühl-Zürbes P., Drassner C., Daniel C., Klewer M., Steinkasserer A. (2015). L Particles Transmit Viral Proteins from Herpes Simplex Virus 1-Infected Mature Dendritic Cells to Uninfected Bystander Cells, Inducing CD83 Downmodulation. J. Virol..

[83] Heilingloh C.S., Krawczyk A. (2017). Role of L-Particles during Herpes Simplex Virus Infection. Front. Microbiol..

[84] Cheng H., Wang L., Yang B., Li D., Wang X., Liu X., Tian N., Huang Q., Feng R., Wang Z. (2020). Cutting Edge: Inhibition of Glycogen Synthase Kinase 3 Activity Induces the Generation and Enhanced Suppressive Function of Human IL-10(+) FOXP3(+)-Induced Regulatory T Cells. J. Immunol..

[85] Bi P., Wang J., Lu J., Liu X., Wang F., Luo Y.F., Peng S.D., Li X.P. (2017). Correlation analysis of the Epstein-Barr virus recruited regulatory T cells in nasopharyngeal carcinoma of immune escape. Zhonghua Er Bi Yan Hou Tou Jing Wai Ke Za Zhi.

[86] Meckes D.G., Gunawardena H.P., Dekroon R.M., Heaton P.R., Edwards R.H., Ozgur S., Griffith J.D., Damania B., Raab-Traub N. (2013). Modulation of B-cell exosome proteins by gamma herpesvirus infection. Proc. Natl. Acad. Sci. USA.

[87] Bloom D.C., Kielian M., Maramorosch K., Mettenleiter T.C. (2016). Chapter Two—Alphaherpesvirus Latency: A Dynamic State of Transcription and Reactivation. Advances in Virus Research.

[88] McCarthy K.M., Tank D.W., Enquist L.W. (2009). Pseudorabies virus infection alters neuronal activity and connectivity in vitro. PLoS Pathog..

[89] Laval K., Maturana C.J., Enquist L.W. (2020). Mouse Footpad Inoculation Model to Study Viral-Induced Neuroinflammatory Responses. J. Vis. Exp..

[90] Laval K., Van Cleemput J., Vernejoul J.B., Enquist L.W. (2019). Alphaherpesvirus infection of mice primes PNS neurons to an inflammatory state regulated by TLR2 and type I IFN signaling. PLoS Pathog..

[91] Wang F., Wu J.-M., Zhou Y.-B., Li S.-Y., Cheng Y.-Z., Qi M., Tang W., Wang H., Liu J., Yu H. (2007). Mechanisms of vascular endothelial cell injury induced by human cytomegalovirus. J. Shandong Univ. (Health Sci.).

[92] Zhang T., Ma C., Zhang Z., Zhang H., Hu H. (2021). NF-κB signaling in inflammation and cancer. MedComm (2020).

[93] Zeng Q., Meng Y., LI Y. (2023). Research advances in cytomegalovirus hepatitis. J. Clin. Hepatol..

[94] Da Cunha T., Wu G.Y. (2021). Cytomegalovirus Hepatitis in Immunocompetent and Immunocompromised Hosts. J. Clin. Transl. Hepatol..

[95] Hong J., Zhong L., Liu L., Wu Q., Zhang W., Chen K., Wei D., Sun H., Zhou X., Zhang X. (2023). Non-overlapping epitopes on the gHgL-gp42 complex for the rational design of a triple-antibody cocktail against EBV infection. Cell Rep. Med..

[96] Townsend S., Finlay W.J., Hearty S., O’Kennedy R. (2006). Optimizing recombinant antibody function in SPR immunosensing. The influence of antibody structural format and chip surface chemistry on assay sensitivity. Biosens. Bioelectron..

[97] Singh A., Pasha S.K., Manickam P., Bhansali S. (2016). Single-domain antibody based thermally stable electrochemical immunosensor. Biosens. Bioelectron..

[98] Monson E.A., Lloyd M.G., Johnson R.I., Caracciolo K., Whan J., Rau T.F., Londrigan S.L., Moffat J.F., Mayfosh A.J., Helbig K.J. (2025). GS-1 blocks entry of herpes viruses and more broadly inhibits enveloped viruses. Antiviral Res..

[99] Cohen J.I., Abendroth A., Arvin A.M., Moffat J.F. (2010). The Varicella-Zoster Virus Genome. Varicella-Zoster Virus.

[100] Duus K.M., Grose C. (1996). Multiple regulatory effects of varicella-zoster virus (VZV) gL on trafficking patterns and fusogenic properties of VZV gH. J. Virol..

[101] Zhao G.X., Bu G.L., Liu G.F., Kong X.W., Sun C., Li Z.Q., Dai D.L., Sun H.X., Kang Y.F., Feng G.K. (2023). mRNA-based Vaccines Targeting the T-cell Epitope-rich Domain of Epstein Barr Virus Latent Proteins Elicit Robust Anti-Tumor Immunity in Mice. Adv. Sci..

[102] Liu H., Chen H., Liu Z., Le Z., Nie T., Qiao D., Su Y., Mai H., Chen Y., Liu L. (2020). Therapeutic nanovaccines sensitize EBV-associated tumors to checkpoint blockade therapy. Biomaterials.

[103] Zhong L., Zhang W., Liu H., Zhang X., Yang Z., Wen Z., Chen L., Chen H., Luo Y., Chen Y. (2024). A cocktail nanovaccine targeting key entry glycoproteins elicits high neutralizing antibody levels against EBV infection. Nat. Commun..

[104] Vanarsdall A.L., Chin A.L., Liu J., Jardetzky T.S., Mudd J.O., Orloff S.L., Streblow D., Mussi-Pinhata M.M., Yamamoto A.Y., Duarte G. (2019). HCMV trimer- and pentamer-specific antibodies synergize for virus neutralization but do not correlate with congenital transmission. Proc. Natl. Acad. Sci. USA.

[105] Xie Z., Song Y. (2023). Research strategies and advances in vaccines against human cytomegalovirus infection. Prog. Microbiol. Immunol..

[106] Hu X., Karthigeyan K.P., Herbek S., Valencia S.M., Jenks J.A., Webster H., Miller I.G., Connors M., Pollara J., Andy C. (2024). Human Cytomegalovirus mRNA-1647 Vaccine Candidate Elicits Potent and Broad Neutralization and Higher Antibody-Dependent Cellular Cytotoxicity Responses Than the gB/MF59 Vaccine. J. Infect. Dis..

[107] Johnston C., Gottlieb S.L., Wald A. (2016). Status of vaccine research and development of vaccines for herpes simplex virus. Vaccine.

[108] Spicknall I.H., Looker K.J., Gottlieb S.L., Chesson H.W., Schiffer J.T., Elmes J., Boily M.C. (2019). Review of mathematical models of HSV-2 vaccination: Implications for vaccine development. Vaccine.

[109] Stanberry L.R., Spruance S.L., Cunningham A.L., Bernstein D.I., Mindel A., Sacks S., Tyring S., Aoki F.Y., Slaoui M., Denis M. (2002). Glycoprotein-D–Adjuvant Vaccine to Prevent Genital Herpes. N. Engl. J. Med..

[110] Pražák V., Mironova Y., Vasishtan D., Hagen C., Laugks U., Jensen Y., Sanders S., Heumann J.M., Bosse J.B., Klupp B.G. (2024). Molecular plasticity of herpesvirus nuclear egress analysed in situ. Nat. Microbiol..

[111] Sun A., Wang X., Yang S., Liu Y., Zhang G., Zhuang G. (2021). A rapid and accurate method for herpesviral gnome editing. Chin. J. Biotechnol..

[112] Wu H., Li T., Zeng M., Peng T. (2012). Herpes simplex virus type 1 infection activates the Epstein-Barr virus replicative cycle via a CREB-dependent mechanism. Cell Microbiol..

[113] Botting R.A., Rana H., Bertram K.M., Rhodes J.W., Baharlou H., Nasr N., Cunningham A.L., Harman A.N. (2017). Langerhans cells and sexual transmission of HIV and HSV. Rev. Med. Virol..

[114] Meier A.F., Tobler K., Michaelsen K., Vogt B., Henckaerts E., Fraefel C. (2021). Herpes Simplex Virus 1 Coinfection Modifies Adeno-associated Virus Genome End Recombination. J. Virol..

[115] Meier A.F., Tobler K., Leisi R., Lkharrazi A., Ros C., Fraefel C. (2021). Herpes simplex virus co-infection facilitates rolling circle replication of the adeno-associated virus genome. PLoS Pathog..

[116] Ahmadivand S., Soltani M., Shokrpoor S., Rahmati-Holasoo H., El-Matbouli M., Taheri-Mirghaed A. (2020). Cyprinid herpesvirus 3 (CyHV-3) transmission and outbreaks in Iran: Detection and characterization in farmed common carp. Microb. Pathog..

